# Lowndes County (Miss.) Medical Association

**Published:** 1871-10

**Authors:** W. L. Lipscomb, J. W. M. Shattuck

**Affiliations:** President; Secretary


### LOWNDES COUNTY (Miss.) MEDICAL ASSOCIATION.

The Lowndes County (Miss.) Medical Association met pursuant to adjournment, at 11 o'clock, A. M., June 24th, 1871.

The President, Dr. Lipscomb, being absent, Dr. Ervin occupied the chair.

After calling the roll the minutes of the preceding meeting were read and adopted.

The essayist for the meeting, Dr. Mayo, then read an interesting and exhaustive paper on Puerperal Peritonitis, giving a full and complete history of this formidable disease—discussed the treatment as laid down by standard authorities. In treating this fever, he relies upon quinine, given as follows: twenty grains every two hours until eighty grains are taken. He reported several cases thus treated with success, and recommends this remedy in that disease, which so often ends fatally.

The Essay was discussed by Drs. Price, Franklin, Browning, Vaughan and Maxwell.

Under the head of new business, Dr. Vaughan read an elaborate and interesting Essay on the Thermometer in Disease. Also, read a well considered paper on Periodical days or crises in various diseases. Dr. Vaughan gave some useful data in regard to the temperature of the human body. The normal temperature ranges between 97.3° and 98.3°, and it is found to be nearly the same in cold climates—there is always a slight increase after regular meals; the mean temperature is shortly before the main meal—the maximum is about four hours after—the minimum is at night.

The temperature of the body in sickness varies considerably from that of health—its maximum, by which life may be sustained is nearly 108°, the minimum 81°. In fevers the temperature ranges from 100.5° to 107°.

As a general rule the pulse increases in frequency correspondly with the increase of temperature, and the temperature in nearly all diseases rises higher the nearer the disease draws to a fatal conclusion.

The temperature in disease rises and falls rapidly. Again, in the chilly stage of intermittent fever, the temperature rises 2° above the normal standard.

At the close of the Essay Dr. Vaughan exhibited a thermometer designed for the use of physicians. In his paper on critical days, the Doctor remarked:

First. That a well-treated disease is cured without a crisis.

Second. When our patient recovers, with well marked days of crisis, we have missed the case and the pxtient recovered without benefit from our remedies.

Third. When a crisis appears, and the patient gets worse and worse in spite of our remedies, then we may feel assured we have missed the mark, and may have done actual harm by our medication.

Dr. Price reported a case of severe wound of the abdomen, in a patient who was suffering from intermittant fever. He applied cold water dressing to the wound and gave twenty grains of quinine to prevent return of the chill; repeated the dose in three hours. The patient had no return of chills, neither did he have any fever or peritonitis, but made a rapid and perfect recovery, the wound healing by first intention.

Under miscellaneous business, Dr. Vaughan suggested that the members of the Association subscribe for the leading Medical Journals in different sections of this country and in Europe, these

Journals to be read attentively by the members, and that each volume, when complete, to be the property of the original subscriber. This was discussed by several members, and postponed for further action.

The Secretary offered the following:

*Resolved.* That each Essayist be required to deliver his Essay to the Secretary after it is read or before the next meeting, it shall then become the property of the Association. The Secretary will take charge of all the Essays and hold them subject to the order of the Association.

The resolution was adopted.

Dr. Brownlee was appointed by the chair, as the Essayist for the next regular meeting.

There being no further business before the meeting, the Association adjourned.

W. L. LIPSCOMB, M. D., *President.*

J. W. M. SHATTUCK, *Secretary.*

